# Supplementary material for: The Construction and Exploration of a Comprehensive MicroRNA Centered Regulatory Network in Foxtail Millet (Setaria italica L.)
Source: Front Plant Sci. 2022 May 6;13:848474. doi: 10.3389/fpls.2022.848474 (PMC9121102; doi:10.3389/fpls.2022.848474)
Supplement: Supplementary file 3 [file Data_Sheet_3.zip › Sit-miR168_Seita.7G201100.1_664_TPlot.pdf]

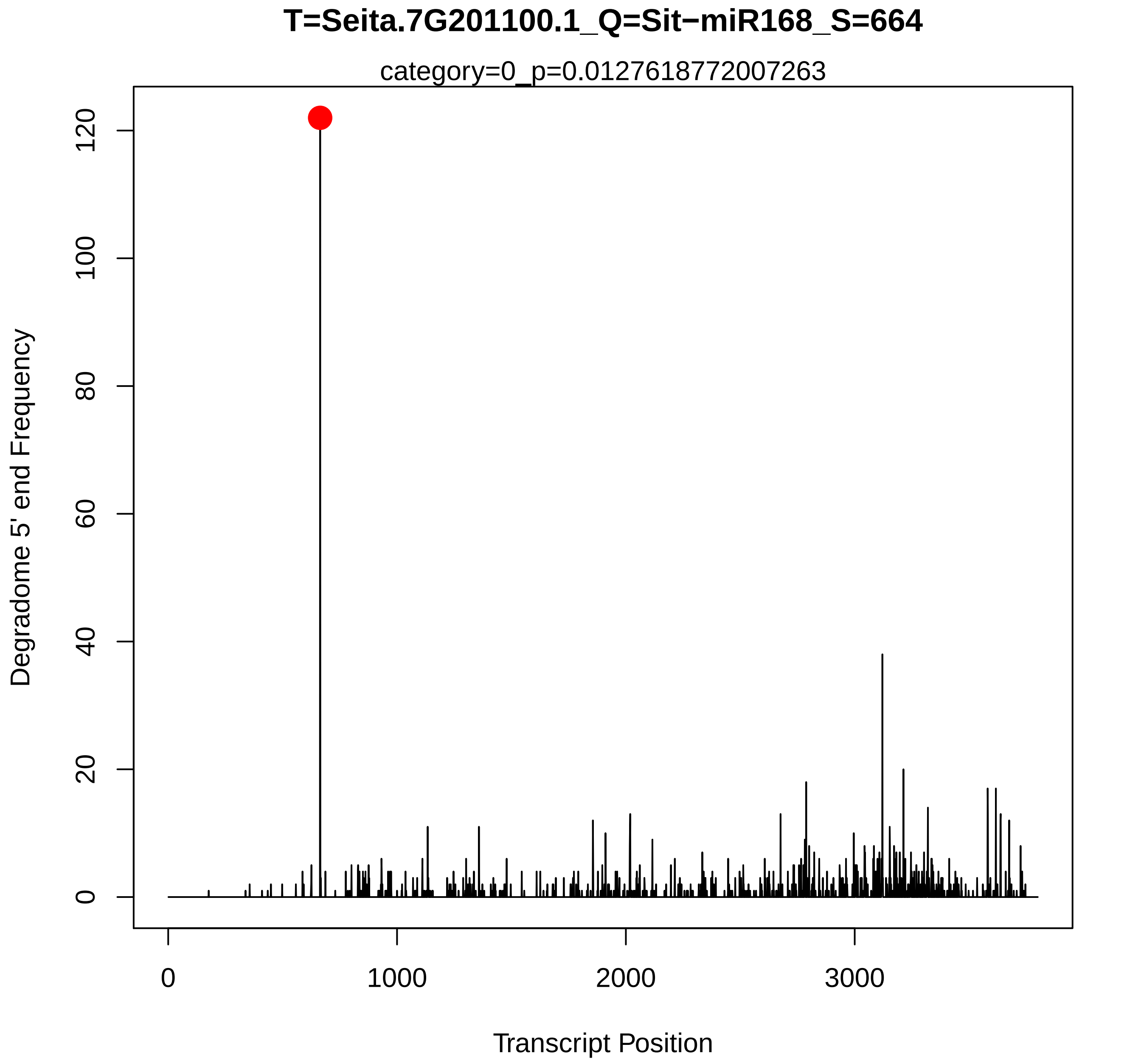
T=Seita.7G201100.1_Q=Sit−miR168_S=664
category=0_p=0.0127618772007263
Degradome 5' end Frequency
Transcript Position
0
20
40
60
80
100
120
0
1000
2000
3000
